# Sex-Associated Differences in Cytomegalovirus Prevention: Prophylactic Strategy is Potentially Associated With a Strong Kidney Function Impairment in Female Renal Transplant Patients

**DOI:** 10.3389/fphar.2020.534681

**Published:** 2020-12-21

**Authors:** Arturo Blazquez-Navarro, Chantip Dang-Heine, Chris Bauer, Nicole Wittenbrink, Kerstin Wolk, Robert Sabat, Oliver Witzke, Timm H. Westhoff, Birgit Sawitzki, Petra Reinke, Oliver Thomusch, Christian Hugo, Nina Babel, Michal Or-Guil

**Affiliations:** ^1^ Department of Biology, Systems Immunology Lab, Humboldt-Universität zu Berlin, Berlin, Germany; ^2^ Berlin Institute of Health Center for Regenerative Therapies (BCRT), Berlin-Brandenburger Centrum für Regenerative Therapien, Charité–Universitätsmedizin Berlin, Berlin, Germany; ^3^ Center for Translational Medicine, Universitätsklinikum der Ruhr-Universität Bochum, Herne, Germany; ^4^ Clinical Study Center (CSC), Berlin Institute of Health, and Charitét - Universitättsmedizin Berlin, Corporate Member of Freie Universitätt Berlin, Humboldt-Universitätt Zu Berlin, Campus Charitét Mitte Berlin, Germany; ^5^ MicroDiscovery GmbH, Berlin, Germany; ^6^ Department of Dermatology and Allergy, Psoriasis Research and Treatment Center, Institute of Medical Immunology, Charité–Universitätsmedizin Berlin, Berlin, Germany; ^7^ Department of Dermatology and Allergy, Interdisciplinary Group of Molecular Immunopathology, Institute of Medical Immunology, Charité–Universitätsmedizin Berlin, Berlin, Germany; ^8^ Klinik für Infektiologie, Universitätsklinikum Essen, Essen, Germany; ^9^ Berlin Center for Advanced Therapies (BeCAT), Charité–Universitätsmedizin Berlin, Berlin, Germany; ^10^ Klinik für Allgemein- und Viszeralchirurgie, Universitätsklinikum Freiburg, Freiburg, Germany; ^11^ Medizinische Klinik III - Bereich Nephrologie, Universitätsklinikum Carl Gustav Carus, Dresden, Germany; ^12^ Institute of Medical Immunology, Charité–Universitätsmedizin Berlin, Berlin, Germany

**Keywords:** prevention, valganciclovir, gender, graft function, cytomegalovirus

## Abstract

Post-transplantation cytomegalovirus (CMV) syndrome can be prevented using the antiviral drug (val)ganciclovir. (Val)ganciclovir is typically administered following a prophylactic or a pre-emptive strategy. The prophylactic strategy entails early universal administration, the pre-emptive strategy, early treatment in case of infection. However, it is not clear which strategy is superior with respect to transplantation outcome; sex-specific effects of these prevention strategies are not known. We have retrospectively analyzed 540 patients from the multi-centre Harmony study along eight pre-defined visits: 308 were treated according to a prophylactic, 232 according to a pre-emptive strategy. As expected, we observed an association of prophylactic strategy with lower incidence of CMV syndrome, delayed onset and lower viral loads compared to the pre-emptive strategy. However, in female patients, the prophylactic strategy was associated with a strong impairment of glomerular filtration rate one year post-transplant (difference: −11.8 ± 4.3 ml min^−1^·1.73 m^−2^, *p* = 0.006). Additionally, we observed a tendency of higher incidence of acute rejection and severe BK virus reactivation in the prophylactic strategy group. While the prophylactic strategy was more effective for preventing CMV syndrome, our results suggest for the first time that the prophylactic strategy might lead to inferior transplantation outcomes in female patients, providing evidence for a strong association with sex. Further randomized controlled studies are necessary to confirm this potential negative effect.

## Introduction

Cytomegalovirus (CMV) is a herpesvirus often reported as the most important viral pathogen after kidney transplantation (Elfadawy et al., 2013; Le Page et al., 2013; Fehr et al., 2015). It is a major cause of morbidity and mortality, being associated with retinitis, pneumonitis, colitis, encephalitis, allograft damage and allograft loss, among others (Egli et al., 2007; Elfadawy et al., 2013; Le Page et al., 2013; Fehr et al., 2015). CMV syndrome or disease may occur as a consequence of reactivation of latent infections or through primary infection, acquired from the donor or from the environment (Fehr et al., 2015). The major risk factor for CMV syndrome or disease is the pre-transplantation serostatus: CMV seronegative transplant recipients with a seropositive donor (D^+^R^−^) have the highest risk, while seropositive recipients (R^+^) have an intermediate risk and seronegative recipients with seronegative donors (D^−^R^−^) have the lowest risk (Fehr et al., 2015). Moreover, the use of immunosuppressive drugs like rabbit antithymocyte globulin (ATG) can additionally increase the incidence of CMV (re)activations (Malvezzi et al., 2015).

The standards in prevention and treatment of CMV (re)activation are based on ganciclovir or its oral prodrug valganciclovir (KDIGO Transplant Work Group, 2009; Kotton et al., 2018). In addition to antiviral therapy, CMV-specific T cell immunity has been shown to control CMV viral reactivations, determining the outcome of disease (Sester et al., 2001; Bunde et al., 2005; Egli et al., 2008). Two prevention strategies are routinely employed in the clinic: prophylactic and pre-emptive (KDIGO Transplant Work Group, 2009; Fehr et al., 2015; Kotton et al., 2018). The prophylactic strategy is based on the universal administration of (val)ganciclovir for patients with a CMV risk constellation, usually during 3–6 months after transplantation (KDIGO Transplant Work Group, 2009; Kotton et al., 2018). In the pre-emptive strategy, patients are regularly monitored for CMV through quantitative polymerase chain reaction (qPCR) or pp65 antigenemia test; (val)ganciclovir is only administered after a positive test, ideally before any symptoms of CMV syndrome or disease manifest (KDIGO Transplant Work Group, 2009; Kotton et al., 2018). The pre-emptive strategy thus leads to a reduction of unnecessary treatments, which is advantageous with respect to the appearance of side effects and resistances against antiviral drugs (KDIGO Transplant Work Group, 2009; Kotton et al., 2018).

While the KDIGO guideline of 2009 preferred prophylaxis as the standard of prevention, the more recent reference CMV management guideline recommends both strategies for the prevention of CMV disease in patients with both high or intermediate CMV mismatch-based risk constellation (KDIGO Transplant Work Group, 2009; Kotton et al., 2018). However, the differences in outcome with regard to other criteria, including renal function and other viral (re)activations is largely unclear. Interestingly, there is evidence of sex differences in both ganciclovir pharmacokinetics and the anti-CMV immune response (Villacres et al., 2004; Perrottet et al., 2009; Di Benedetto et al., 2015; Hassan et al., 2016; Stranzinger et al., 2016; Fleck-Derderian et al., 2017; Momper et al., 2017; Lindemann et al., 2018). Thus, female patients have been shown to have a faster ganciclovir clearance, and distinct anti-CMV immunological profiles, e.g., higher number of IL-21 secreting anti-CMV T cells (Villacres et al., 2004; Perrottet et al., 2009; Di Benedetto et al., 2015; Momper et al., 2017; Lindemann et al., 2018). In spite of this, there are to our knowledge still no studies on the influence of sex on the clinical outcomes of CMV prevention strategies. In this work, we provide first tentative evidence that prophylaxis might be associated with inferior transplantation outcomes in female patients.

## Materials and Methods

### Patient Population

As part of the systems medicine project e:KID, we conducted a sub-study within the randomized, multi-centre, investigator-initiated Harmony trial (NCT 00724022) (Thomusch et al., 2016; Blazquez-Navarro et al., 2018) to determine the impact of CMV prevention strategy on transplant outcome. For this, CMV, Epstein-Barr virus (EBV) and BK virus (BKV) viral loads, white blood cell count and creatinine were measured at predetermined eight study visits (Blazquez-Navarro et al., 2018). This viral monitoring was non-interventional and centrally performed and was independent from the internal, interventional viral monitoring (see *Patient Monitoring*). The study was carried out in compliance with the Declaration of Helsinki and Good Clinical Practice.

### Patient Medication

According to study design, patients were treated with a quadruple (arm A) or triple (arms B and C) immunosuppressive therapy (Thomusch et al., 2016). Patients in arm A received an induction therapy with basiliximab and maintenance therapy consisting of tacrolimus (Advagraf ®, Astellas), mycophenolate mofetil (MMF) and corticosteroids. Patients in arm B received the same treatment as in arm A, but corticosteroids were withdrawn at day 8. Patients in arm C received the same treatment as in arm B, except induction was achieved with ATG, instead of basiliximab. The study protocol proposed furthermore that patients with a D^+^R^−^ for either CMV or CMV, as well as patients in arm C regardless of their mismatch, should receive a valganciclovir prophylaxis (Thomusch et al., 2016).

### Patient Monitoring

Patients were monitored for transplantation outcomes during the first post-transplantation year. Graft function was monitored along seven visits, scheduled at the second week, first month, second month, third month, sixth month, ninth month, and 12th month. To assess graft function, glomerular filtration rate was calculated using the CKD-EPI formula, measured in mL·min^−1^·1.73 m^−2^ (Levey et al., 2009). Serious adverse events were defined following the Good Clinical Practice guidelines. Suspected episodes of acute rejection had to be confirmed through biopsy; histologic characteristics were described according to the Banff criteria of 2005 (Solez et al., 2007). Regarding the outcome assessment, acute rejection was analyzed excluding borderline rejections. Routine surveillance biopsies were allowed but not mandatory.

### Clinical Monitoring and Management of Clinical Complications

Viral (re)activations were monitored during the first post-transplantation year and managed at local centers as described previously (Blazquez-Navarro et al., 2018). CMV in particular was monitored for all patients, independently of the prevention strategy. Monitoring was performed independently from the above described CMV viral load measurements and was based on three different methods: serum PCR viral load measurements; test for pp65 antigenemia and symptom monitoring according to the internal center standards. Diagnosis of CMV syndrome was likewise based on these methods, where a qPCR over 1,000 copies·mL^−1^ was defined as positive. Patients with CMV syndrome were treated based on internal center standards. Suggested treatment was (val)ganciclovir treatment according to local standards with/or without reduction of tacrolimus and MMF dose. No data on the time point of CMV syndrome diagnostic were available for this study; no data on CMV disease were available.

### Screening of CMV, EBV and BKV Viraemia

In parallel to the clinical monitoring performed at each center, peripheral blood samples from the seven post-transplant visits as well as a pre-transplant visit were centrally monitored for CMV, EBV and BKV by TaqMan qPCR, as described previously (Blazquez-Navarro et al., 2018). The centralized viral load assessment was non-interventional.

### Definition of CMV Prevention Strategy Groups and Characterization of Antiviral Treatments

Patients were stratified into two prevention strategy groups based on the (val)ganciclovir treatments during the first 14 days. All patients that started a (val)ganciclovir treatment during the first 14 days were assigned into the prophylactic strategy group; the rest of the sub-cohort was classified in the pre-emptive strategy group. The 14 days threshold was chosen to allow comparability with our previous prospective study on the topic (VIPP), in which recruiting took place during the first two post-transplant weeks (Witzke et al., 2012; Witzke et al., 2018). CMV syndrome was treated equally for both strategy groups, as explained above. Antiviral treatments with no data on the end time point were considered for the calculation of valganciclovir average daily dose, even though they cannot be included in the calculation of the duration of treatment. The average daily dose refers to the period(s) in which the patient received a valganciclovir treatment, not the entire first post-transplant year. Accordingly, reported MMF dose and tacrolimus concentration/dose (C/D) ratio correspond to the 14 days threshold.

### Viraemia-Based Patient Classification

To assess the efficacy of prevention strategies regarding viral (re)activations, patients were classified based on their peak viral load values for CMV, EBV and BKV, as previously published (Blazquez-Navarro et al., 2018). Briefly, the classifications are defined as follows: “detectable viral load” corresponds to patients with at least one viral load measurement over detection limit (250 copies·mL^−1^) (Blazquez-Navarro et al., 2018), “elevated viral load” to patients with at least one viral load measurement over 2000 copies·mL^−1^, “high viral load” to patients with at least one viral load measurement over 10,000 copies·mL^−1^. These groups overlap with each other.

### Descriptive Statistics and Baseline Analysis

Categorical variables are summarized here as numbers and frequencies; quantitative variables are reported as median and interquartile range (IQR). Differences between the groups were calculated using Pearson’s chi-square test with continuity correction (or two-tailed Fisher’s exact test, when stated); odds ratios (OR) and 95% confidence intervals (95% CI) are provided. In all cases, odds ratio over one denote a higher prevalence of the adverse event in the pre-emptive strategy group. Differences in quantitative variables between groups were analyzed using the two-tailed Mann-Whitney test. Kaplan-Meier curves for time to occurrence of the first CMV (re)activation were calculated using the R survival package (version 2.43–3); strategy groups were compared using the log-rank test. Correlations are reported employing Spearman’s rho and *p* value). Box plots depict the median, first and third quartile of a variable; the maximum length of the whiskers corresponds to 1.5 times the IQR.

In baseline analysis, a *p* value below 0.050 was considered significant. For descriptive statistics, *p* values are reported purely for illustrative reasons – no definition of statistical significance is employed.

### Multi-Parameter Regression Modeling

To determine the influence of prevention strategies, sex and their interaction on transplantation outcomes, we performed multi-parameter regression controlling for confounders: The regression models used for the analysis incorporate as independent variables the prevention strategies, sex and their interaction term, as well as all selected confounding factors, and as dependent variable the outcome of interest. For categorical binary outcomes, logistic regression was employed, for continuous outcomes linear regression was used. The choice of confounders was performed through backward elimination by Akaike’s information criterion starting from a full model, as the criteria for the allocation to a prevention strategy are unknown so that a selection based on medical criteria is not possible. The full model incorporated – apart from prevention strategies, sex and their interaction – all measured demographic factors (see Table 1 and Supplementary Table S1) and the transplantation center. For the analysis of the eGFR one year after transplantation (eGFR-1y), CMV, BKV and EBV peak viral loads and acute rejection were additionally included as potential confounders, as these events preceded or were simultaneous to eGFR-1y and might hence have an influence on it. Peak viral loads were included in the models as log-transformed (base 10); viral loads below detection level were set to zero. All analyses were run on complete cases, without performing imputation. Thus, the multiparameter analysis of viral reactivation and eGFR-1y was performed on patients with available measurements one year after transplantation.

**TABLE 1:** Differences in patient baseline characteristics between strategy groups.

Variable		Prophylactic strategy group (*N* = 308)	Pre-emptive strategy group (*N* = 232)	*p* value
Female sex		104 (33.8%)	90 (38.8%)	0.265
Caucasian race		304 (98.7%)	231 (99.6%)	0.397^a^
Recipient age (years)		55 [46–64]	57 [44–64]	0.988
Body mass index (kg·m^−2^)		26.3 [23.5–29.7]	25.4 [22.8–28.4]	0.059
CMV mismatch-based risk	High (D^+^R^−^)	119 (39.1%)	27 (12.1%)	<0.001
	Medium (R^+^)	137 (45.1%)	129 (57.8%)	
	Low (D^−^R^−^)	48 (15.8%)	67 (30.0%)	
EBV mismatch-based risk	High (D^+^R^−^)	13 (5.1%)	11 (6.2%)	0.583^a^
	Medium (R^+^)	239 (93.4%)	161 (91.0%)	
	Low (D^−^R^−^)	4 (1.6%)	5 (2.8%)	
Donor age (years)		55 [48–65]	55 [46–65]	0.931
No previous transplantations		298 (96.8%)	216 (94.7%)	0.346
Living donor		31 (10.1%)	35 (15.4%)	0.088
Expanded criteria donor		136 (44.2%)	99 (42.7%)	0.798
High donor serum creatinine (>1.5 mg dL^−1^)		35 (11.4%)	39 (16.8%)	0.090
Cold ischemia time (min)		626 [427–844]	600 [414–840]	0.505
Number of HLA A, B and DR mismatches		3 [2–4]	3 [1–4]	0.457
Panel-reactive antibodies before transplantation		23 (7.6%)	17 (7.7%)	1.000
White blood cell count (cells·L^−1^)		7.2 [5.7–8.9]	7.1 [6.0–8.5]	0.676
Therapy arm	A (basiliximab + steroids)	93 (30.2%)	96 (41.4%)	<0.001
	B (basiliximab)	92 (29.9%)	83 (35.8%)	
	C (ATG)	123 (39.9%)	53 (22.8%)	
Low MMF daily dose (<2000 mg·day^−1^)		37 (12%)	45 (19.4%)	0.025
Tacrolimus C/D level (ng·mL^−1^·mg^−1^·kg·day)		65.6 [43.6–99.2]	63.7 [42.9–92.2]	0.341

a Data are given as number (percentage) or median [interquartile range]. Expanded criteria donors are defined as follows: age over 60 years or age over 50 years and at least two of the following factors: cerebrovascular accident as the cause of death, hypertension or a serum creatinine level over 1.5 mg dL^−1^. p value is calculated based on Pearson’s chi-square test or Fisher’s exact test for binary variables (marked with^a^) and based on Mann-Whitney test for continuous variables. Data on the cause of end-stage kidney disease are summarized in Supplementary Table S1.

After backward elimination, the resulting model for each outcome was tested for multi-collinearity and (in the case of linear regression) for homoscedasticity: Multi-collinearity was assessed calculating the generalized variance-inflation factor, with a threshold of five to exclude a factor. Homoscedasticity was evaluated with the studentized Breusch-Pagan test; if it cannot be assumed (*p* < 0.050), robust standard errors are reported. The resulting model for each outcome is provided in the Supplementary Table S2. The *p* values for the independent variables of the final model were calculated employing the *t* test.

In the multi-parameter analysis, a *p* value below 0.050 was considered significant. *p* values were not corrected for multiple testing, as this study was of exploratory nature (Bender and Lange, 2001; Velentgas et al., 2013; Li et al., 2017).

## Results

### Definition of Study Sub-cohorts

To assess the effects of CMV prevention strategy on transplantation outcome, we retrospectively analyzed the cohort of an existent study (N = 540 patients from 18 centers) with a female ratio of 35.9% (N = 194) (Thomusch et al., 2016; Blazquez-Navarro et al., 2018). Patients were grouped into two sub-cohorts, based on whether they started an antiviral therapy during the first two post-transplant weeks (prophylactic strategy group, N = 308) or not (pre-emptive strategy group, N = 232) (see *Definition of CMV Prevention Strategy Groups and Characterization of Antiviral Treatments*). As described previously, viral load (CMV, EBV and BKV), graft function and other clinical markers were collected along eight visits during the first post-transplant year; a total of 3,715 blood samples were analyzed (Blazquez-Navarro et al., 2018).

In this work, we have evaluated the effects of prevention strategy and sex on the main outcome eGFR-1y and the secondary outcomes, incidence of acute rejection, CMV complications, and BKV and EBV (re)activations. The analyses were performed based on the following approach: For descriptive purposes, single-parameter differences between sub-cohorts were assessed; multi-parameter regression analysis – controlling for all potential confounders – was employed to determine any effects of prevention strategy, sex and their interaction on transplantation outcomes. After an assessment of the baseline characteristics of the sub-cohorts, we describe in detail the most important findings in the next sections.

### Study Sub-cohorts Characteristics

To identify differences at baseline between the two prevention strategy sub-cohorts regarding demographics or treatment procedures, we performed comparative statistics (see Table 1, for cause of end-stage kidney disease see Supplementary Table S1). As shown in Table 1, significant (*p* < 0.050) differences were found for MMF daily dose, CMV mismatch-based risk and therapy arm; the difference was highly significant (*p* < 0.001) for the latter two factors; specifically among female patients a significant difference in body mass index and CMV mismatch was found, but not in therapy arm nor MMF daily dose (see Supplementary Table S3). The differences in CMV risk and therapy arm are in conformance with the study protocol; the MMF dose was significantly associated with the different transplantation centers (Kruskal-Wallis’ *p* = 0.002).

59 patients (25.4%) of the pre-emptive strategy group were treated with (val)ganciclovir after the second post-transplantation week. In total, 367 patients (68.0%) received (val)ganciclovir during the first post-transplant year, independently of their prevention strategy group; use of antivirals in both groups is shown in Table 2. As expected, valganciclovir treatments in patients of the pre-emptive strategy cohort were significantly shorter and had significantly higher daily doses than in the prophylactic strategy group. The latter is in conformance with current guidelines, which recommend for pre-emptive treatments of CMV viral load a valganciclovir dose twice as high as for prophylaxis (Kotton et al., 2018).

**TABLE 2:** Antiviral treatment details for the two strategy groups.

Variable	Prophylactic strategy group (*N* = 308)	Pre-emptive strategy group treated with (val)ganciclovir (*N* = 59)	*p* value
Median time under (val)ganciclovir (days)	118 [87–182]	92 [46–155]	0.006
Valganciclovir average daily dose (mg·day^−1^)	277 [165–450]	450 [205–454]	<0.001
Patients treated with intravenous ganciclovir	32 (10.4%)	8 (13.6%)	0.623

Data are given as number (percentage) or median [interquartile range].

Regarding differences in antiviral treatment or outcomes between female and male patients, we observed no differences between sexes (Table 3).

**TABLE 3:** Differences between sexes in treatment characteristics and outcomes of the first post-transplantation year.

Variables		Male patients (*N* = 346)	Female patients (*N* = 194)	*p* value
Treatment characteristics				
Prophylactic strategy group		204 (59%)	104 (53.6%)	0.265
Pre-emptive strategy group treated with (val)ganciclovir		40 (11.6%)	19 (9.8%)	0.626
Median time under (val) ganciclovir (days)		113 [84–177]	107 [85–181]	0.868
valganciclovir average daily dose (mg·day^−1^)		328 [187–450]	362 [193–450]	0.619
Patients treated with intravenous ganciclovir		27 (7.8%)	13 (6.7%)	1.000
Outcomes				
Serious adverse event		204 (59.0%)	121 (62.4%)	0.493
eGFR-1y		48.2 [36-5–61.4]	46.8 [33.3–58.8]	0.341
Acute rejection		38 (11.0%)	22 (11.3%)	1.000
CMV	Detectable viral load	57 (16.5%)	35 (18.0%)	0.730
	Elevated viral load	24 (6.9%)	15 (7.7%)	0.866
	High viral load	13 (3.8%)	5 (2.6%)	0.629
	Syndrome	78 (22.5%)	35 (18.0%)	0.261
BKV	Detectable viral load	173 (50.0%)	87 (44.8%)	0.289
	Elevated viral load	76 (22.0%)	45 (23.2%)	0.825
	High viral load	41 (11.8%)	18 (9.3%)	0.438
EBV	Detectable viral load	74 (21.4%)	35 (18.0%)	0.414
	Elevated viral load	28 (8.1%)	9 (4.6%)	0.178
	High viral load	9 (2.6%)	2 (1.0%)	0.343^a^

a Data are given as number (percentage) or median [interquartile range]. p value is calculated based on Pearson’s chi-square test or Fisher’s exact test for binary variables (marked with^a^) and on Mann-Whitney test for continuous variables. For the definition of (re)activation severity degrees see Viraemia-Based Patient Classification. As it can be observed, there were no differences between sexes with respect to (val)ganciclovir treatments and the measured outcomes.

### Prophylactic Strategy Group Was Associated With a Serious Impairment of Graft Function in Female Patients

A descriptive analysis showed that patients in the prophylactic strategy group had, in general, a poorer transplantation course than those in the pre-emptive strategy group, with a higher incidence of total serious adverse events (64.6% vs. 54.3%, *p* = 0.020, OR: 0.65 [0.45–0.94]). For the main outcome, renal function, single-parameter analysis likewise revealed a difference between the prevention groups. Thus, eGFR-1y was lower in the prophylactic strategy group compared to the pre-emptive group (45.6 [33.5–58.3] vs. 50.3 [38.1–64.5] ml·min^−1^.1.73 m^−2^, *p* = 0.011). Of note, the difference in eGFR was noticeable for all visits from the third post-transplant month on (Supplementary Figure S1).

**FIGURE 1:**
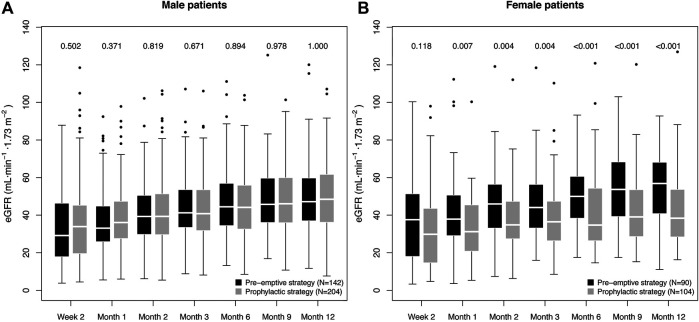
Box plot of the graft function dynamics of the prevention strategy groups stratified for sex. The numbers indicate the *p* value of the difference in eGFR between the prevention strategy groups, as calculated using the Mann-Whitney test. The *p* values for the first six measurements are included only to facilitate understanding on the eGFR dynamics, and are therefore not adjusted for multiple testing.

Importantly, the impairment of eGFR-1y in the prophylactic group was only observed for female patients, with a difference of 18.5 ml min^−1^·1.73 m^−2^ (38.4 [28.8–53.6] vs. 56.8 [41.3–67.9] ml min^−1^.1.73 m^−2^, *p* < 0.001). Among male patients, the prophylactic strategy group had a slightly higher median eGFR-1y (48.5 [36.3–61.5] vs. 47.2 [37.2–59.6] ml min^−1^.1.73 m^−2^, *p* = 1.000). A difference in eGFR for females could be observed already one month after transplantation (Figure 1).

Multi-parameter regression incorporating all potential confounders confirmed a significant, strong association of the interaction term prophylactic strategy:female sex with decreased eGFR-1y (estimate: −11.8 ± 4.3 ml min^−1^.1.73 m^−2^, *p* = 0.006); while no significant association was found for prevention strategy (*p* = 0.651) or sex alone (*p* = 0.138). For more details on the multi-parameter regression model, see Supplementary Table S2A. As a tendency toward lower eGFR in females under the prophylactic strategy (see Figure 1B) was already observed two weeks after transplantation (eGFR-2w), we tested additionally the possibility of a difference in baseline conditions as the cause for the observed association. Therefore, we repeated the analysis incorporating eGFR-2w as a confounder (Supplementary Table S2B). Remarkably, in spite of the highly significant correlation of eGFR-2w with eGFR-1y (0.45 ± 0.04, *p* < 0.001), the negative effect of prophylactic strategy:female sex was consistently strong (−10.4 ± 3.7 ml min^−1^.1.73 m^−2^, *p* = 0.005). We additionally repeated the analysis excluding patients with a D^−^R^-^ CMV constellation (N = 115), finding a similar effect in spite of the reduction in patient number (−9.8 ± 4.0 ml min^−1^.1.73 m^−2^, *p* = 0.016; see Supplementary Table S2C). In conclusion, we did not find any evidence of spuriousness of the observed effect of prophylactic strategy:female sex on the eGFR-1y.

We further investigated the nature of the difference in eGFR between prevention strategies in female patients, examining the associations of daily dose and beginning of therapy with eGFR-1y. We did not observe any negative effect of high daily doses: We compared the female patients in the pre-emptive strategy group that received a valganciclovir treatment, with those in the prophylactic strategy group, as the first group had a higher daily dose than the second (*p* = 0.041). Thus, we observed that these patients had a higher eGFR-1y than those in the prophylactic group (38.4 [28.8–53.6] vs. 57.7 [40.1–66.6] ml·min^−1^.1.73 m^−2^, *p* = 0.005), in spite of the higher valganciclovir dose. On the other hand, we observed an effect of therapy timing in eGFR-1y, with a positive correlation between day of treatment beginning and eGFR-1y (*ρ* = 0.27, *p* = 0.015) among female patients who received (val)ganciclovir.

### The Prophylactic Strategy Was Associated With Significantly Lower CMV Viral Loads and Incidence of Syndrome Than the Pre-emptive Strategy

We further evaluated the effectivity of the strategies in the prevention of CMV complications. The single-parameter, descriptive analysis showed a higher incidence of CMV viral load in the pre-emptive strategy group (19.8% vs. 14.9%, *p* = 0.167); for CMV syndrome a higher incidence was found in the prophylactic strategy group (see Supplementary Table S4A). The latter was not unexpected, as most patients with high CMV risk were in the prophylactic strategy group (see Table 2). Stratifying for CMV risk, a clear trend for lower incidence of CMV (re)activation was observed in the prophylactic strategy group; but not for CMV syndrome (Supplementary Table S4B). However, the results of the multi-parameter regression (Supplementary Tables S2D,E) show that prophylactic strategy had a significant association with both lower peak CMV viral load (-0.63 ± 0.20 log_10_ (copies·mL^−1^), *p* = 0.002) and CMV syndrome incidence (-1.45 ± 0.63, *p* = 0.020). No significant sex effects, nor interactions between prevention strategy and sex were observed for these outcomes.

**FIGURE 2:**
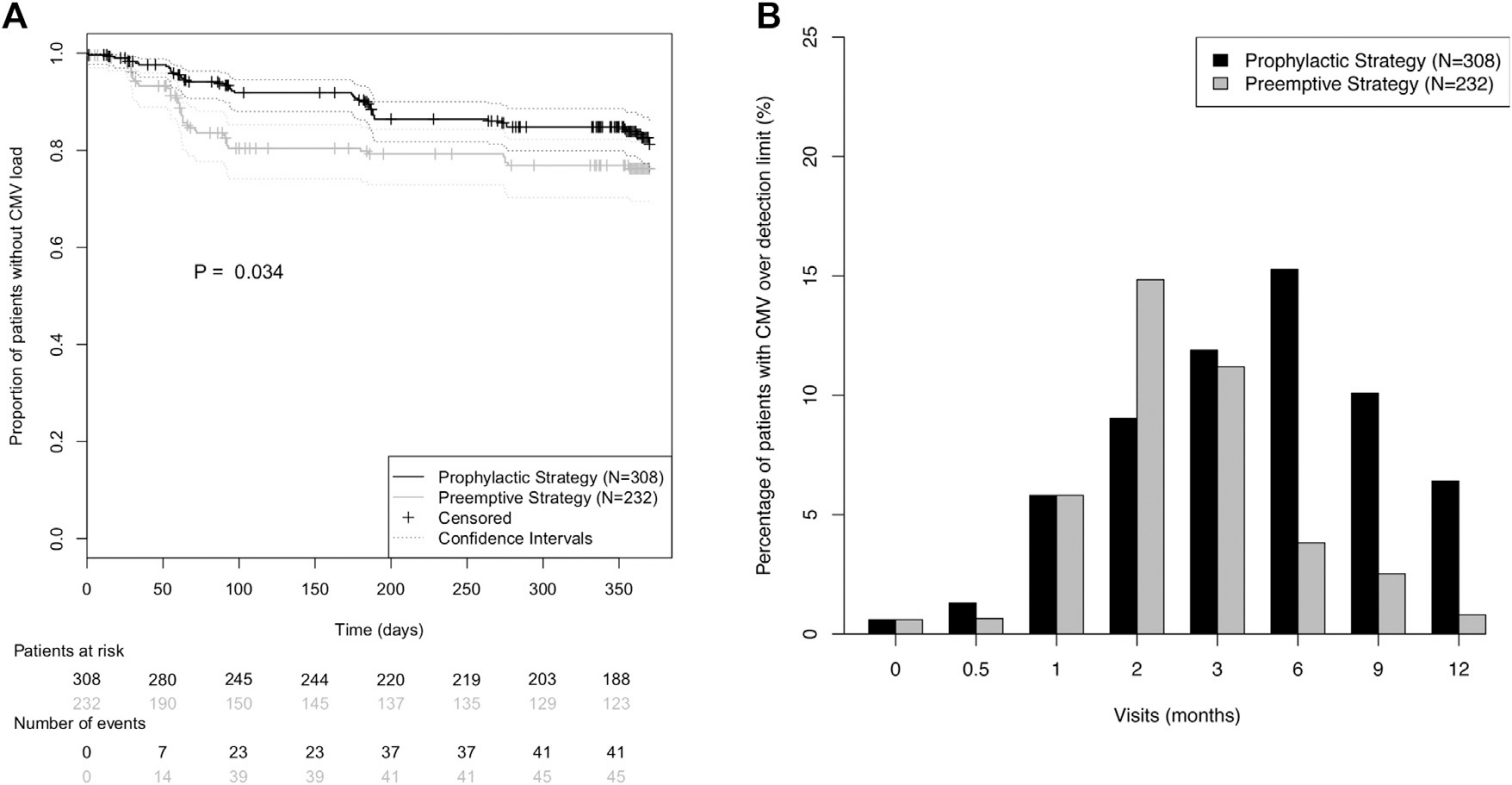
Incidence of CMV (re)activation in the prevention strategy groups during the first post-transplant year. **(A)** Kaplan-Meier curves for absence of CMV (re)activation during the first post-transplant year. CMV (re)activation was defined as viral load over detection limit. Prevention strategy groups were compared using the log-rank test. **(B)** Prevalence of CMV viral load over detection limit for each of the eight protocol visits.

Interestingly, CMV incidence showed different temporal patterns in the two strategy groups (Figure 2): While in the pre-emptive strategy group 86.7% of all CMV load events occurred in the first 100 days post-transplant, in the prophylactic strategy group it was only 56.1% (Figure 2A). Moreover, a higher prevalence of detectable CMV viral load was observed in the prophylactic strategy group for all study visits after the third month (Figure 2B).

### There Was a Tendency Toward Higher Incidence of Acute Rejection and BKV Reactivation Among Patients Under Prophylactic Strategy

Regarding the important complication acute rejection, a tendency toward higher incidence was found in the prophylactic strategy group (14.9% vs. 6.0%, *p* = 0.002, OR: 0.37 [0.18–0.70]). However, the multi-parameter analysis (Supplementary Table S2F) could not confirm this tendency, as only a borderline significant association of prophylactic strategy group with rejection (−0.83 ± 0.51, *p* = 0.100) was observed; there was no evidence of an effect of sex, nor of the interaction with prevention strategy.

Regarding the effects of prevention strategy for other viruses, no evidence of an effect of prevention strategy was found for EBV, neither through (stratified) single-parameter analysis (Supplementary Tables S4A,C), nor through multi-parameter analysis (Supplementary Table S2G). Furthermore, there was no evidence of sex-specific effects. On the other hand, we found a higher incidence of severe BKV (re)activation in patients of the prophylactic strategy group (*p* = 0.056, OR: 0.55 [0.29–1.01]), see Supplementary Table S4A. The multi-parameter analysis (Supplementary Table S2H) showed likewise a borderline significant association of prophylactic strategy with higher BKV viral loads (0.51 ± 0.29 log_10_ (copies·mL^−1^), *p* = 0.084), while no effect of sex could be observed.

## Discussion

The goal of our study was to evaluate the clinical efficacy and sex-associated differences of two common CMV prevention strategies in a large cohort of kidney transplant patients from the multi-centre Harmony study. The main finding of the study is the first tentative evidence suggesting superiority of the pre-emptive strategy in female patients with respect to graft function (Small et al., 2005; Khoury et al., 2006; Kliem et al., 2008; Spinner et al., 2010; Van Der Beek et al., 2010; Witzke et al., 2012; Florescu et al., 2014; Meije et al., 2014; Witzke et al., 2018). The observed effect was very large, corresponding to an increase of 11.8 ± 4.3 ml min^−1^.1.73 m^−2^ in eGFR-1y. This is especially relevant, as eGFR-1y is an accepted marker for long term transplantation outcomes (Kasiske et al., 2011). Even after controlling for differences in eGFR observed two weeks after transplantation (at a time point in which effects of prevention strategy are already thinkable), the effect of prevention strategy among female patients remained consistently strong. However, due to the inherent limitations of a retrospective, non-randomized study, the interpretation of this potential effect requires caution.

Interestingly, our results highlight the importance of sex-associated effects in transplantation. In recent years, sex differences have emerged as an essential factor in clinical studies (Rásky et al., 2017). In transplantation, several complications are associated with sex, including acute rejection, graft loss and viral (re)activations (Meier-Kriesche et al., 2001; Hirsch et al., 2013; Momper et al., 2017; Lindemann et al., 2018). However, the underlying reasons for these sex differences are not well understood; possible causes include the hormonal regulation of the immune system, the effects of pregnancy, and differences in the metabolism of drugs routinely employed in transplantation (Momper et al., 2017). For example, there is tentative evidence of sex-related differences in the pharmacokinetics of (val)ganciclovir (Perrottet et al., 2009; Momper et al., 2017). Thus, ganciclovir clearance has been observed to be 24% faster in female transplantation patients, suggesting higher activity of the organic anion transporter 1 (Perrottet et al., 2009; Momper et al., 2017). Furthermore, it has been shown repeatedly that women and men have different anti-CMV immunological profiles: (Villacres et al., 2004; Di Benedetto et al., 2015; Lindemann et al., 2018) Thus, Lindemann et al. have observed that these different immunological profiles may have an influence on the graft function, as they observed an association of high numbers of IL-21-secreting anti-CMV T cells with both female sex and lower eGFR in a clinical transplantation context (Lindemann et al., 2018). While none of these two observations provide a direct explanation for the observed effect of (val)ganciclovir prophylaxis on eGFR-1y, they demonstrate that further sex-associated differences for pharmacokinetics and pharmacodynamics, anti-CMV immune response and their consequences on renal function are conceivable.

Our analyses may provide some evidence on the nature of the hypothetical negative effect of prophylactic strategy on eGFR-1y. Although the impaired graft function in the female prophylactic strategy group can be partly explained through the higher incidence of BKV severe (re)activation and rejection, the results of the multi-parameter analysis suggest an independent association of prevention strategy with graft function, regardless of these adverse events (Salvadori et al., 2006; Schwarz et al., 2012; Blazquez-Navarro et al., 2018). Therefore, our results do not support the hypothesis that these adverse events are the main cause for the difference in eGFR-1y between sub-cohorts. Regarding possible nephrotoxic effects of the antiviral drug, we did not find any association of higher valganciclovir doses with lower eGFR–rather, the opposite association was observed – in contrast to Heldenbrand et al (Heldenbrand et al., 2016). The absence of a negative dose-dependent effect suggests that the observed difference was not a consequence of nephrotoxicity of valganciclovir. On the other hand, the time of beginning of the (val)ganciclovir therapy could be determinant for the eGFR-1y: We observed a positive correlation between time of beginning of (val)ganciclovir treatment and eGFR (i.e. the later patients began the therapy, the higher the renal function). This correlation could also explain the positive association of antiviral dose with eGFR-1y, as patients receiving a higher valganciclovir dose as part of a pre-emptive treatment had later treatments compared to the prophylactic strategy.

Albeit being highly speculative, we hypothesize that the observed results may be (at least in part) caused by an immunological mechanism. As we demonstrated in a previous study, an increased number of CMV-specific T-cells upon CMV (re)activation is associated with reduced alloreactivity and improved graft function in renal transplantation patients (Nickel et al., 2009). Similarly, in liver transplantation, primary CMV infection has been found to be associated with donor-specific CD8^+^ T-cell hyporesponsiveness and increased Vδ1/Vδ2 γδ T-cell ratio – a surrogate marker for operational tolerance (Shi et al., 2015). Accordingly, the higher rate of asymptomatic CMV (re)activation found in the pre-emptive strategy group could potentially lead to regulatory γδ T-cell-based graft protection and explain the better graft function and lower incidence of acute rejection. Therefore, an early administration of (val)ganciclovir would potentially hinder the development of this protective immune response. Importantly, this hypothesis does not contradict the commonly encountered association between CMV and acute rejection, which was in fact also observed in our patient cohort (Blazquez-Navarro et al., 2018). Rather, it suggests a direction for this effect, with acute rejection or its treatment as a cause of CMV reactivation, rather than a consequence. This in line with the literature, since the hypothetical negative effects of CMV on acute rejection remain highly controversial, while the opposite has been observed in several recent studies (Dickenmann et al., 2001; Erdbruegger et al., 2012; Feng et al., 2016; Felipe et al., 2019; Jorgenson et al., 2019).

Our hypothesis would also explain the observed positive correlation between day of beginning of antiviral therapy and renal function, as according to this premise only the early administration would have negative consequences on the building of the protective immune response. This hypothesis is compatible with the observed differences between male and female recipients, as sex-associated differences in the anti-CMV immunity have been shown to correlate with graft function (Lindemann et al., 2018). This observation shows how sex and anti-CMV immunity could potentially interact and affect eGFR (Lindemann et al., 2018). Therefore, further research, including systems medicine approaches, is needed to better understand the effects of CMV prevention strategies from an immune, virological and pharmacokinetic point of view – with emphasis on sex-associated differences–and their effects on transplantation outcome (Maier et al., 2017; Blazquez-Navarro et al., 2018).

Of interest, the prophylactic strategy group had a higher incidence of late-onset CMV (from the sixth month on); such increases of viral (re)activation incidence after the end of prophylaxis have been observed before (Khoury et al., 2006; Florescu et al., 2014). We also observed a tendency toward higher incidence of rejection and severe BK virus (re)activation among patients in the prophylactic strategy group, although these tendencies could not be confirmed through multi-parameter analysis. While we have already reported a negative effect of prophylaxis on rejection within the VIPP study–albeit only for the D^−^R^+^ subgroup–this study would be the first to suggest such an association in the entire cohort (Witzke et al., 2012; Witzke et al., 2018). Regarding BKV, the observed pattern is in line with three recent studies (Reischig et al., 2015; Reischig et al., 2018; Reischig et al., 2019). This effect could be explained through the increased incidence of acute rejection among patients in the prophylactic strategy group, as episodes of acute rejection and anti-rejection treatment have been associated with BKV reactivation (Schold et al., 2009; Borni-Duval et al., 2013).

On the other hand, we did not observe any effect of prevention strategy on EBV (re)activation. This is relevant, as there is currently no consensus in the literature on this topic. Even though a number of publications have observed an effect against EBV or post-transplant lymphoproliferative disease (the main EBV-associated complication), a meta-study with 2,366 participants saw no effect of prophylaxis for this EBV complication (Funch et al., 2005; Aldabbagh et al., 2016; Cameron et al., 2017).

This study is based on the prospective Harmony study, a trial designed with the goal of identifying which immunosuppressive drug combination is superior with respect to acute rejections and secondary to a number of other outcome variables, including graft function and viral (re)activations (Thomusch et al., 2016). A shortcoming of the present study is the fact that prevention strategy groups were not randomized, and no power calculation was performed with respect to this question. Even though we have controlled for all measured demographic factors in the analyses and other potential confounding factors–including the first measured eGFR after transplantation–we cannot exclude bias, for example in unmeasured factors such as sex-mismatch, as the cause of the observed differences. A further limitation is related to the criteria employed for deciding the prevention strategy for each patient: As the decision to adopt a prophylactic or a pre-emptive strategy was taken by each individual physician or center, it is difficult to ascertain the background, especially if the decision differs from what would be expected based on the study protocol. This could potentially introduce unknown bias in the use of prevention strategies. On the other hand, our study does have some advantages: We have analyzed a larger (N = 540) and more heterogeneous cohort (patients with all CMV mismatch-based risk constellations) than most studies on the matter, thereby achieving higher statistical power (Khoury et al., 2006; Kliem et al., 2008; Spinner et al., 2010; Van Der Beek et al., 2010; Witzke et al., 2012; Florescu et al., 2014; Witzke et al., 2018). Moreover, our study design could be viewed as closer to clinical reality, with similar valganciclovir doses and prophylaxis duration to those routinely employed in the clinic (Rissling et al., 2018). Based on the limitations of the study, we deem our results as evidence that further research is needed to determine the effects of prevention strategies on transplantation outcome and their hypothetical interactions with sex.

In summary, our study provides the first tentative evidence in the literature suggesting superiority of the pre-emptive approach in female patients. Even though the prophylactic strategy was associated with reduced prevalence of CMV (re)activation and syndrome, we also found a potential association with a strong renal function impairment. The effects of prevention strategy on graft function were found in the multi-parameter analysis to be independent from all potential confounders for which there were available data. Moreover, we observed tentative evidence of a sex-independent tendency toward higher incidence of acute rejection and BKV (re)activation. Importantly, further studies are needed to confirm these observations: Based on our results, randomized controlled studies comparing pre-emptive and prophylactic strategy for the end-point eGFR-1y–stratifying for sex–are paramount. Moreover, observational studies analyzing the potential relationship between time of beginning of the valganciclovir therapy and the renal function could further shed light on the matter.

## Data Availability Statement

The datasets for this article are not publicly available because the patients have not consented to the publication of their data. Requests for limited access to the data should be directed to NB, (nina.babel@charite.de).

## Ethics Statement

The study was carried out in compliance with the Declaration of Helsinki and Good Clinical Practice. All participants provided written informed consent prior to inclusion into the study. The trial was approved by the Ethics Committee of the Universitätsklinikum Carl Gustav Carus Dresden. The trial is registered with ClinicalTrials.gov, number NCT00724022 (https://clinicaltrials.gov/ct2/show/study/NCT00724022). Date of registration was July 2008 (retrospectively registered), date of enrollment was June 2008.

## Author Contributions

NB, NW, CH, OT, CB, KW, RS, OW, TW, BS, PR, and MO-G contributed to the study design, sample collection, and/or sample management. CD-H carried out experiments. AB-N, NB, and MO-G performed data interpretation. AB-N, NB, and MO-G drafted the manuscript. All authors have contributed to the manuscript and approved the final version of the manuscript for submission.

## Funding

This work was funded by the German Federal Ministry of Education and Research (BMBF), project e:KID (01ZX1312), and by the European Regional Development Fund (ERDF), project SepsisDataNet.NRW. We acknowledge support from the German Research Foundation (DFG) and the Open Access Publication Fund of Charité — Universitätsmedizin Berlin. The funders had no role in data collection, data analysis, data interpretation, writing of the manuscript, or manuscript submission.

## Conflict of Interest

OW has received research grants for clinical studies, speaker's fees, honoraria and travel expenses from Amgen, Astellas, Bristol-Myers Squibb, Chiesi, Janssen-Cilag, MSD, Novartis, Roche, Pfizer, and Sanofi. OW is supported by an unrestricted grant of the Rudolf-Ackermann-Stiftung (Stiftung für Klinische Infektiologie).

The remaining authors declare that the research was conducted in the absence of any commercial or financial relationships that could be construed as a potential conflict of interest.
