# Supplementary material for: Sex-Associated Differences in Cytomegalovirus Prevention: Prophylactic Strategy is Potentially Associated With a Strong Kidney Function Impairment in Female Renal Transplant Patients
Source: Front Pharmacol. 2020 Dec 21;11:534681. doi: 10.3389/fphar.2020.534681 (PMC7845412; doi:10.3389/fphar.2020.534681)
Supplement: Supplementary file 1 [file table1.pdf]

Cause of end-stage kidney disease	Prophylactic strategy group (N=308)	Pre-emptive strategy group (N=232)	P Value
Hypertension or large vessel disease	111 (36.0%)	87 (37.8%)	0.738
Glomerulonephritis	79 (25.6%)	69 (30.0%)	0.308
Polycystic kidney disease (adult type, dominant)	64 (20.8%)	38 (16.5%)	0.256
Diabetes	32 (10.4%)	23 (10.0%)	0.997
Interstitial nephritis or pyelonephritis	20 (6.5%)	19 (8.3%)	0.539
Secondary glomerulonephritis or vasculitis	7 (2.3%)	4 (1.7%)	0.765[a]
Other hereditary or congenital diseases	11 (3.6%)	8 (3.5%)	1.000
Neoplasms or tumours	4 (1.3%)	0 (0.0%)	0.139[a]
Other	109 (35.4%)	72 (31.3%)	0.368
Undefined cause	27 (9.3%)	24 (11.0%)	0.621

Table S1 – Differences in cause of end-stage kidney disease between strategy groups. Data are given in number (percentage). P value is calculated based on Pearson's chi-square test or Fisher's exact test (marked with [a]). Causes of end-stage kidney disease are not mutually exclusive.
