# Supplementary material for: Sex-Associated Differences in Cytomegalovirus Prevention: Prophylactic Strategy is Potentially Associated With a Strong Kidney Function Impairment in Female Renal Transplant Patients
Source: Front Pharmacol. 2020 Dec 21;11:534681. doi: 10.3389/fphar.2020.534681 (PMC7845412; doi:10.3389/fphar.2020.534681)
Supplement: Supplementary file 2 [file table2.pdf]

Explanatory variables	Estimate	Standard error	P value
(Intercept)	104.7251	7.2251	<0.001
Prophylactic strategy	-1.1111	2.4547	0.651
Female sex	4.7712	3.2039	0.138
Prophylactic strategy:Female sex	-11.7902	4.27531	0.006
Recipient age (years)	-0.2050	0.0938	0.030
Donor age (years)	-0.4254	0.0731	<0.001
Body mass index ($kg{\cdot}m^{-2}$)	-0.8207	0.2174	<0.001
No panel-reactive antibodies before transplantation	-6.1990	3.5829	0.085
Cause of end-stage renal disease: Diabetes	6.4232	3.9054	0.101
Tacrolimus C/D ratio ($ng{\cdot}mL^{-1}{\cdot}mg^{-1}{\cdot}kg{\cdot}day$)	0.0415	0.0169	0.015
Acute rejection	-8.1700	3.3870	0.017
Peak BKV viral load ($copies{\cdot}mL^{-1}$)	-0.8291	0.5276	0.117

Table S2A – Multivariate analysis of prevention strategy and sex effects on eGFR-1y. eGFR-1y was estimated by linear regression. Confounders were selected employing backwards elimination, starting with all demographic factors (Table 1 and Table S1), CMV, BKV and EBV peak viral loads, acute rejection and transplantation centre. The results for the regression are shown for prevention strategy, sex, their interaction and all selected confounders under explanatory variables. The regression analysis was performed on data of 257 patients.

BK virus (BKV), Concentration/dose (C/D), Estimated glomerular filtration rate one year after transplantation (eGFR-1y)

Explanatory variables	Estimate	Standard error	P value
(Intercept)	63.6352	4.9268	<0.001
Prophylactic strategy	-1.1104	2.1201	0.601
Female sex	3.4447	2.7492	0.211
Prophylactic strategy:Female sex	-10.4247	3.6624	0.005
eGFR-2w	0.4481	0.0440	<0.001
Recipient age (years)	-0.1891	0.0796	0.018
Donor age (years)	-0.3491	0.0632	<0.001
White blood cell count (cells$\cdot L^{-1}$)	-0.0029	0.0018	0.120
Tacrolimus C/D ratio (ng$\cdot mL^{-1}\cdot mg^{-1}\cdot$kg$\cdot$day)	0.0263	0.0145	0.070
Acute rejection	-5.1438	2.9289	0.080
Peak BKV viral load (copies$\cdot mL^{-1}$)	-0.8396	0.4560	0.067

Table S2B – Multivariate analysis of prevention strategy and sex effects on eGFR-1y controlling for eGFR-2w. eGFR-1y was estimated by linear regression. Confounders were selected employing backwards elimination, starting with all demographic factors (Table 1 and Table S1), CMV, BKV and EBV peak viral loads, acute rejection and transplantation centre. The results for the regression are shown for prevention strategy, sex, their interaction and all selected confounders under explanatory variables. The regression analysis was performed on data of 257 patients.

BK virus (BKV), Concentration/dose (C/D), Estimated glomerular filtration rate two weeks after transplantation (eGFR-2w), Estimated glomerular filtration rate one year after transplantation (eGFR-1y)

Explanatory variables	Estimate	Standard error	P value
(Intercept)	53.0172	4.3360	<0.001
Prophylactic strategy	-0.7669	2.2985	0.739
Female sex	2.7482	3.1542	0.385
Prophylactic strategy:Female sex	-9.8481	4.0320	0.016
eGFR-2w	0.4705	0.0481	<0.001
Donor age (years)	-0.3738	0.0611	<0.001
Tacrolimus C/D ratio ($ng \cdot mL^{-1} \cdot mg^{-1} \cdot kg \cdot day$)	0.0222	0.0147	0.133
Peak BKV viral load ($copies \cdot mL^{-1}$)	-0.9225	0.4878	0.060

Table S2C – Multivariate analysis of prevention strategy and sex effects on eGFR-1y controlling for eGFR-2w and excluding patients with a D^-R^- CMV constellation. eGFR-1y was estimated by linear regression. Confounders were selected employing backwards elimination, starting with all demographic factors (Table 1 and Table S1), CMV, BKV and EBV peak viral loads, acute rejection and transplantation centre. The results for the regression are shown for prevention strategy, sex, their interaction and all selected confounders under explanatory variables. The regression analysis was performed on data of 202 patients.

BK virus (BKV), Concentration/dose (C/D), Estimated glomerular filtration rate two weeks after transplantation (eGFR-2w), Estimated glomerular filtration rate one year after transplantation (eGFR-1y)

Explanatory variables	Estimate	Standard error	P value
(Intercept)	1.4494	0.2198	<0.001
Prophylactic strategy	-0.6303	0.2037	0.002
Female sex	-0.10267	0.262407	0.696
Prophylactic strategy:Female sex	-0.1147	0.3458	0.740
CMV mismatch-based risk: Medium (R^+)	-0.3015	0.1953	0.124
CMV mismatch-based risk: Low (D^-R^-)	-1.0877	0.2365	<0.001
Cause of end-stage renal disease: Other hereditary or congenital diseases	1.3197	0.4976	0.008
White blood cell count (cells·L^{-1})	0.0004	0.0002	0.041

Table S2D – Multivariate analysis of prevention strategy and sex effects on CMV peak viral load. Peak viral load in logarithmic scale (with a value of 0 for viral load below detection limit) was estimated by linear regression. Confounders were selected employing backwards elimination, starting with all demographic factors (Table 1 and Table S1) and transplantation centre. The results for the regression are shown for prevention strategy, sex, their interaction and all selected confounders under explanatory variables. The regression analysis was performed on data of 263 patients.

Cytomegalovirus (CMV), Seronegative donor and seronegative recipient (D^-R^-), Seropositive Recipient (R^+)

Explanatory variables	Estimate	Standard error	P value
(Intercept)	1.8760	1.5080	0.213
Prophylactic strategy	-1.4530	0.6254	0.020
Female sex	-0.2526	0.7072	0.721
Prophylactic strategy:Female sex	1.1730	0.9099	0.197
Donor age (years)	0.0371	0.0139	0.007
Body mass index (kg·m^{-2})	-0.0741	0.0459	0.106
CMV mismatch-based risk: Medium (R^{+})	-1.0470	0.4996	0.036
CMV mismatch-based risk: Low (D^{-}R^{-})	-5.3100	1.2910	<0.001
Living donor	-3.0450	0.9367	0.001
Cause of end-stage renal disease: Hypertension	-0.8922	0.5935	0.133
Cause of end-stage renal disease: Polycystic kidney disease (adult type, dominant)	-1.0430	0.5179	0.044
Cause of end-stage renal disease: Diabetes	2.2570	0.7601	0.003
Cause of end-stage renal disease: Undefined cause	-1.4950	0.7735	0.053
White blood cell count (cells·L^{-1})	0.0035	0.0633	0.956
Tacrolimus C/D ratio (ng·mL^{-1}·mg^{-1}·kg·day)	0.0074	0.0036	0.038
Centre effects	-	-	<0.001

Table S2E – Multivariate analysis of prevention strategy and sex effects on CMV syndrome. Occurrence of CMV syndrome during the first post-transplantation year was estimated by logistic regression. Confounders were selected employing backwards elimination, starting with all demographic factors (Table 1 and Table S1) and transplantation centre. The P value for centre effects refers to the minimum P value of any transplantation centre. The results for the regression are shown for prevention strategy, sex, their interaction and all selected confounders under explanatory variables. The regression analysis was performed on data of 263 patients.

Cytomegalovirus (CMV), Concentration/dose (C/D), Seronegative donor and seronegative recipient (D^{-}R^{-}), Epstein-Barr virus (EBV), Seropositive Recipient (R^{+})

Explanatory variables	Estimate	Standard error	P value
(Intercept)	-3.3267	0.9108	<0.001
Prophylactic strategy	0.8339	0.5068	0.100
Female sex	0.4419	0.6373	0.488
Prophylactic strategy:Female sex	-0.6604	0.7748	0.394
Number of HLA A, B and DR mismatches	0.3970	0.1135	<0.001
No previous transplantations	-1.2087	0.7184	0.093
Cause of end-stage renal disease: Polycystic kidney disease (adult type, dominant)	1.1388	0.4606	0.013
Cause of end-stage renal disease: Interstitial nephritis or pyelonephritis	1.0950	0.5887	0.063
Cause of end-stage renal disease: Other	1.0006	0.4159	0.016

Table S2F – Multivariate analysis of prevention strategy and sex effects on acute rejection. Occurrence of acute rejection during the first post-transplantation year was estimated by logistic regression. Confounders were selected employing backwards elimination, starting with all demographic factors (Table 1 and Table S1) and transplantation centre. The results for the regression are shown for prevention strategy, sex, their interaction and all selected confounders under explanatory variables. The regression analysis was performed on data of 347 patients.

Explanatory variables	Estimate	Standard error	P value
(Intercept)	2.0121	0.6328	0.002
Prophylactic strategy	-0.0661	0.2110	0.754
Female sex	0.1978	0.2590	0.446
Prophylactic strategy:Female sex	-0.3712	0.344279	0.282
Number of HLA A, B and DR mismatches	0.0906	0.0537	0.093
Donor with expanded criteria	0.2935	0.1790	0.103
No panel-reactive antibodies before transplantation	-0.7575	0.3121	0.016
No previous transplantation	-0.7250	0.4808	0.133
Cause of end-stage renal disease: Hypertension	0.3788	0.2049	0.066
Cause of end-stage renal disease: Polycystic kidney disease (adult type, dominant)	-0.6266	0.2186	0.005
Cause of end-stage renal disease: Diabetes	-0.7404	0.3183	0.021
Cause of end-stage renal disease: Neoplasms or tumours	2.0199	0.9449	0.034
Cause of end-stage renal disease: Other	-0.3452	0.1825	0.060
Cause of end-stage renal disease: Undefined cause	-0.5238	0.2792	0.062
Low MMF daily dose (< 2000 $mg \cdot day^{-1}$)	-0.0004	0.0002	0.060
Centre effects	-	-	0.005

Table S2G – Multivariate analysis of prevention strategy and sex effects on EBV peak viral load. Peak viral load in logarithmic scale (with a value of 0 for viral load below detection limit) estimated by linear regression. Confounders were selected employing backwards elimination, starting with all demographic factors (Table 1 and Table S1) and transplantation centre. The P value for centre effects refers to the minimum P value of any transplantation centre. The results for the regression are shown for prevention strategy, sex, their interaction and all selected confounders under explanatory variables. The regression analysis was performed on data of 263 patients.

Epstein-Barr virus (EBV), Mycophenolate mofetil (MMF)

Explanatory variables	Estimate	Standard error	P value
(Intercept)	1.7718	0.2742	<0.001
Prophylactic strategy	0.5100	0.2944	0.084
Female sex	-0.2990	0.3531	0.398
Prophylactic strategy:Female sex	-0.2240	0.4792	0.641
Number of HLA A, B and DR mismatches	-0.1321	0.0704	0.062
Cause of end-stage renal disease: Interstitial nephritis or pyelonephritis	0.8906	0.4643	0.056
Cause of end-stage renal disease: Other	0.5334	0.2551	0.038

Table S2H – Multivariate analysis of prevention strategy and sex effects on BKV peak viral load. Peak viral load in logarithmic scale (with a value of 0 for viral load below detection limit) estimated by linear regression. Confounders were selected employing backwards elimination, starting with all demographic factors (Table 1 and Table S1) and transplantation centre. The results for the regression are shown for prevention strategy, sex, their interaction and all selected confounders under explanatory variables. The regression analysis was performed on data of 263 patients.

BK virus (BKV)
