# Supplementary material for: Sex-Associated Differences in Cytomegalovirus Prevention: Prophylactic Strategy is Potentially Associated With a Strong Kidney Function Impairment in Female Renal Transplant Patients
Source: Front Pharmacol. 2020 Dec 21;11:534681. doi: 10.3389/fphar.2020.534681 (PMC7845412; doi:10.3389/fphar.2020.534681)
Supplement: Supplementary file 3 [file table3.pdf]

Variable		Females in prophylactic strategy group (N=104)	Females in pre-emptive strategy group (N=90)	P value
Caucasian race		104 (100.0%)	90 (100.0%)	-
Recipient age (years)		57 [47-65]	59 [48-64]	0.910
Body mass index (kg·m^{-2})		26.5 [23.5-30.9]	24.5 [21.9-27.9]	0.005
CMV mismatch -based risk	High (D^+R^-)	36 (35%)	8 (9.3%)	<0.001
	Medium (R^+)	55 (53.4%)	55 (64.0%)	
	Low (D^-R^-)	12 (11.7%)	23 (26.7%)	
EBV mismatch -based risk	High (D^+R^-)	2 (2.4%)	3 (4.4%)	0.373[a]
	Medium (R^+)	82 (96.5%)	62 (91.2%)	
	Low (D^-R^-)	1 (1.2%)	3 (4.4%)	
Donor age (years)		59 [48-69]	56 [45-64]	0.183
No previous transplantations		101 (97.1%)	85 (95.5%)	0.705[a]
Living donor		14 (13.5%)	15 (17.0%)	0.625
Expanded criteria donor		57 (54.8%)	37 (41.1%)	0.078
High donor serum creatinine (>1.5 mg·dL^{-1})		11 (10.6%)	11 (12.2%)	0.893
Cold ischaemia time (min)		621 [419-882]	656 [421-863]	0.949
Number of HLA A, B and DR mismatches		3 [2-4]	3 [1-4]	0.087
Panel-reactive antibodies before transplantation		12 (11.8%)	9 (10.5%)	0.961
White blood cell count (cells·L^{-1})		7.5 [6.0-9.2]	7.2 [6.1-9.3]	0.655
Therapy arm	A (basiliximab+steroids)	28 (26.9%)	35 (38.9%)	0.106
	B (basiliximab)	35 (33.7%)	31 (34.4%)	

	C (ATG)	41 (39.4%)	24 (26.7%)	
Low MMF daily dose (< 2000 mg·day^{-1})		17 (16.3%)	16 (17.8%)	0.942
Tacrolimus C/D level (ng·mL^{-1}·mg^{-1}·kg·day)		60.9 [41.9-98.1]	58.6 [41.3-79.6]	0.417
Cause of end-stage kidney disease	Hypertension or large vessel disease	31 (29.8%)	35 (38.9%)	0.238
	Glomerulonephritis	19 (18.3%)	26 (28.9%)	0.115
	Polycystic kidney disease (adult type, dominant)	29 (27.9%)	14 (15.6%)	0.059
	Diabetes	11 (10.6%)	4 (4.4%)	0.185
	Interstitial nephritis or pyelonephritis	11 (10.6%)	14 (15.6%)	0.414
	Secondary glomerulonephritis or vasculitis	2 (1.9%)	2 (2.2%)	1.000[a]
	Other hereditary or congenital diseases	6 (5.8%)	3 (3.3%)	0.508[a]
	Neoplasms or tumours	0 (0.0%)	0 (0.0%)	-
	Other	31 (29.8%)	29 (32.2%)	0.836
	Undefined cause	9 (9.3%)	6 (7.1%)	0.803

Table S3 – Differences in patient baseline characteristics between strategy groups within the female sub-cohort. Data are given as number (percentage) or median [interquartile range]. Expanded criteria donors are defined as follows: age over 60 years or age over 50 years and at least two of the following factors: cerebrovascular accident as the cause of death, hypertension or a serum creatinine level over 1.5 mg·dL^{-1}. P value is calculated based on Pearson's chi-square test or Fisher's exact test for binary variables (marked with [a]) and based on Mann-Whitney test for continuous variables.
