# Supplementary material for: Sex-Associated Differences in Cytomegalovirus Prevention: Prophylactic Strategy is Potentially Associated With a Strong Kidney Function Impairment in Female Renal Transplant Patients
Source: Front Pharmacol. 2020 Dec 21;11:534681. doi: 10.3389/fphar.2020.534681 (PMC7845412; doi:10.3389/fphar.2020.534681)
Supplement: Supplementary file 4 [file table4.pdf]

Viral Event	Severity	Prophylactic strategy group (N=308)	Pre-emptive strategy group (N=232)	P value	OR [95% CI]
CMV (re)activation	Detectable viral load	46 (14.9%)	46 (19.8%)	0.167	1.41 [0.87-2.27]
	Elevated viral load	19 (6.2%)	20 (8.6%)	0.357	1.43 [0.71-2.92]
	High viral load	10 (3.2%)	8 (3.4%)	1.000	1.06 [0.36-3.05]
CMV syndrome	All	78 (25.3%)	35 (15.1%)	0.005	0.52 [0.33-0.83]
	Mild	39 (50.0%)	24 (68.6%)	0.204	-
	Moderate	31 (39.7%)	9 (25.7%)		
	Severe	8 (10.3%)	2 (5.7%)		
EBV (re)activation	Detectable viral load	62 (20.1%)	47 (20.3%)	1.000	1.01 [0.64-1.57]
	Elevated viral load	17 (5.5%)	20 (8.6%)	0.215	1.61 [0.78-3.37]
	High viral load	6 (1.9%)	5 (2.2%)	1.000[a]	1.11 [0.26-4.42]
BKV (re)activation	Detectable viral load	157 (51.0%)	103 (44.4%)	0.153	0.77 [0.54-1.10]
	Elevated viral load	78 (25.3%)	43 (18.5%)	0.077	0.67 [0.43-1.04]
	High viral load	41 (13.3%)	18 (7.8%)	0.056	0.55 [0.29-1.01]

Table S4A – Differences in viral events between strategy groups. Data are given in number (percentage). P value is calculated based on Pearson's chi-square test or Fisher's exact test (marked with [a]). Odds ratio and confidence intervals are given only for viral events that were significant different between the strategy groups. For the definition of (re)activation severity degrees see Methods (2.7). With respect to severity of CMV syndrome, the percentage refers to the total number of CMV syndromes.

BK virus (BKV), Cytomegalovirus (CMV), 95% Confidence interval (95% CI), Epstein-Barr virus (EBV), Odds ratio (OR).

CMV risk constellation	CMV event	Severity	Prophylactic strategy group (N=308)	Pre-emptive strategy group (N=232)	P value	OR [95% CI]
D^+R^- (N=146)	(Re)activation	Detectable	31 (26.1%)	9 (33.3%)	0.598	1.42 [0.51-3.75]
		Elevated	13 (10.9%)	6 (22.2%)	0.123[a]	2.31 [0.65-7.48]
		High	6 (5.0%)	3 (11.1%)	0.368[a]	2.34 [0.35-11.9]
	Syndrome	All	43 (36.1%)	8 (29.6%)	0.677	0.75 [0.26-1.97]
		Mild	14 (32.6%)	6 (75.0%)	0.062[a]	-
		Moderate	22 (51.2%)	1 (12.5%)		
		Severe	7 (16.3%)	1 (12.5%)		
R^+ (N=266)	(Re)activation	Detectable	12 (8.8%)	35 (27.1%)	<0.001	3.86 [1.84-8.63]
		Elevated	5 (3.6%)	13 (10.1%)	0.066	2.95 [0.95-10.88]
		High	3 (2.2%)	5 (3.9%)	0.490[a]	1.80 [0.34-11.81]
	Syndrome	All	30 (21.9%)	27 (20.9%)	0.966	0.94 [0.5-1.77]
		Mild	23 (76.7%)	18 (66.7%)	0.460[a]	-
		Moderate	7 (23.3%)	8 (29.6%)		
		Severe	0 (0.0%)	1 (3.7%)		
D^-R^- (N=115)	(Re)activation	Detectable	2 (4.2%)	1 (1.5%)	0.570[a]	0.35 [0.01-6.94]
		Elevated	0 (0.0%)	1 (1.5%)	1.000[a]	-
		High	0 (0.0%)	0 (0.0%)	-	-
	Syndrome	All	3 (6.2%)	0 (0.0%)	0.070	0.00 [0.00-1.70]
		Mild	2 (66.7%)	0 (0.0%)	1.000[a]	-
		Moderate	0 (0.0%)	0 (0.0%)		
		Severe	1 (33.3%)	0 (0.0%)		
Arm C (N=176)	(Re)activation	Detectable	21 (17.1%)	9 (17%)	1.000	0.99 [0.37-2.49]
		Elevated	10 (8.1%)	5 (9.4%)	0.774[a]	1.18 [0.30-4.02]
		High	5 (4.1%)	2 (3.8%)	1.000[a]	0.93 [0.09-5.88]
	Syndrome	All	29 (23.6%)	8 (15.1%)	0.287	0.58 [0.21-1.43]
		Mild	14 (48.3%)	4 (50.0%)	0.878[a]	-
		Moderate	10 (34.5%)	2 (25.0%)		
		Severe	5 (17.2%)	2 (25.0%)		

Table S4B – Differences in cytomegalovirus complications between strategy groups stratified for CMV risk constellation. Data are given in number (percentage). P value is calculated based on Pearson's chi-square test or Fisher's exact test (marked with [a]). Odds ratio and confidence intervals are given only for viral events that were significant different between the strategy groups. For the

definition of (re)activation severity degrees see Methods (2.7). With respect to severity of CMV syndrome, the percentage refers to the total number of CMV syndromes.
95% Confidence interval (95% CI), Cytomegalovirus (CMV), Seronegative donor and seronegative recipient (D^-R^-), Seropositive donor and seronegative recipient (D^+R^-), Odds ratio (OR), Seropositive Recipient (R^+).

EBV risk constellation	Severity of (re)activation	Prophylactic strategy group (N=308)	Pre-emptive strategy group (N=232)	P value	OR [95% CI]
D^+R^- (N=24)	Detectable	3 (23.1%)	3 (27.3%)	1.000[a]	1.24 [0.13-12.00]
	Elevated	2 (15.4%)	3 (27.3%)	0.630[a]	2.00 [0.18-29.33]
	High	1 (7.7%)	1 (9.1%)	1.000[a]	1.19 [0.01-101.86]
R^+ (N=400)	Detectable	49 (20.5%)	32 (19.9%)	0.979	0.96 [0.56-1.63]
	Elevated	12 (5.0%)	14 (8.7%)	0.209	1.80 [0.75-4.39]
	High	5 (2.1%)	3 (1.9%)	1.000[a]	0.89 [0.14-4.64]
D^-R^- (N=9)	Detectable	1 (25.0%)	1 (20.0%)	1.000[a]	0.77 [0.01-78.24]
	Elevated	0 (0.0%)	0 (0.0%)	-	-
	High	0 (0.0%)	0 (0.0%)	-	-
Arm C (N=176)	Detectable	28 (22.8%)	18 (34%)	0.173	1.74 [0.80-3.73]
	Elevated	8 (6.5%)	6 (11.3%)	0.362[a]	1.83 [0.49-6.39]
	High	2 (1.6%)	3 (5.7%)	0.162[a]	3.60 [0.40-44.31]

Table S4C – Differences in EBV (re)activations between strategy groups stratified for EBV risk constellation. Data are given in number (percentage). P value is calculated based on Pearson's chi-square test or Fisher's exact test (marked with [a]). Odds ratio and confidence intervals are given only for viral events that were significant different between the strategy groups. For the definition of (re)activation severity degrees, see Methods (2.7).

95% Confidence interval (95% CI), Seronegative donor and seronegative recipient (D^-R^-), Seropositive donor and seronegative recipient (D^+R^-), Epstein-Barr virus (EBV), Odds ratio (OR), Seropositive Recipient (R^+).
