# Supplementary figures and images for: Sex-Associated Differences in Cytomegalovirus Prevention: Prophylactic Strategy is Potentially Associated With a Strong Kidney Function Impairment in Female Renal Transplant Patients
Source: Front Pharmacol. 2020 Dec 21;11:534681. doi: 10.3389/fphar.2020.534681 (PMC7845412; doi:10.3389/fphar.2020.534681)

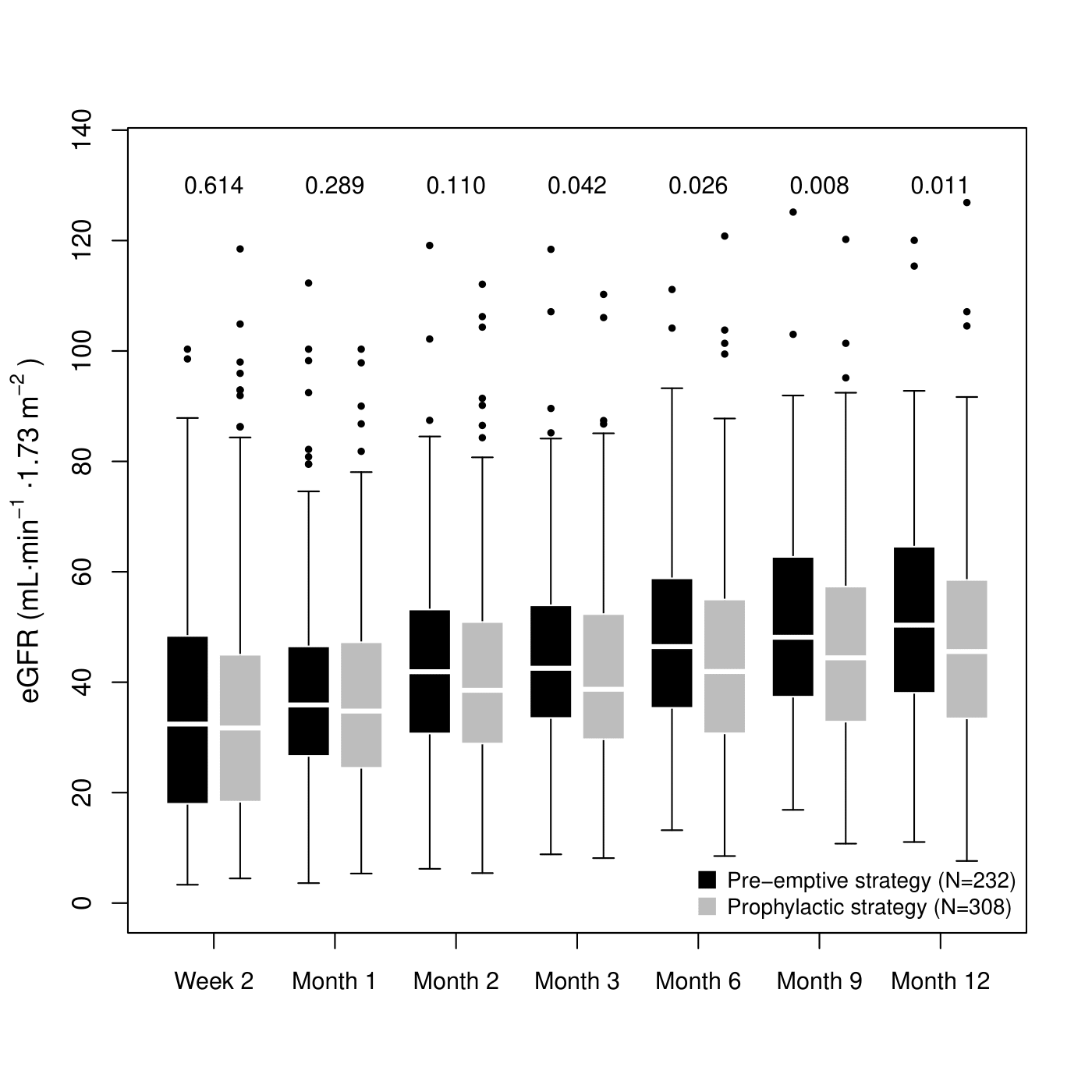

Supplement: Supplementary file 5 [file image1.png]
